# The implementation and effect evaluation of AIDET standard communication health education mode under the King theory of goal attainment: A randomized control study

**DOI:** 10.1097/MD.0000000000036083

**Published:** 2023-12-01

**Authors:** Hao Yang, Wanying Luo, Xue Du, Yujia Guan, Wentao Peng

**Affiliations:** a Department of Medical Genetics Nursing, West China Second University Hospital, Sichuan University/West China School of Nursing, Sichuan University, Chengdu, Sichuan, China; b Key Laboratory of Birth Defects and Related Diseases of Women and Children (Sichuan University), Ministry of Education, Chengdu, Sichuan, China; c Department of Pediatric Kidney Nursing, West China Second University Hospital, Sichuan University, Chengdu, Sichuan, China; d Department of Nursing, West China Second University Hospital, Sichuan University, Chengdu, Sichuan, China.

**Keywords:** AIDET, effect evaluation, health education, nurse–patient communication, standard communication mode, the King theory of goal attainment

## Abstract

**Background:**

This research addresses inadequate understanding of interventional prenatal diagnosis, preoperative anxiety psychological problems in pregnant women undergoing interventional prenatal diagnosis, proposing a health education mode combined AIDET standard communication and King's theory of goal attainment approach to potentially improve health education outcomes, anxiety psychological problems, and patient satisfaction.

**Methods:**

A convenient sampling method was used to select a total of 300 pregnant women who were ready to undergo interventional prenatal diagnosis. They were randomly divided into a implementation group and a control group, with 150 pregnant women in each group. The control group used the communication mode of the traditional process of nurse–patient communication. The implementation group used the AIDET standard communication health education model under the King theory of goal attainment in the process of nurse–patient communication and the interventional prenatal diagnosis health education content questionnaire, the pregnant women’s satisfaction questionnaire, state anxiety scale, and disease uncertainty scale were used for evaluation.

**Results:**

The results of the interventional prenatal diagnosis health education questionnaire, the results of pregnant women’s anxiety, the results of pregnant women’s disease uncertainty, the results of pregnant women’s satisfaction, the implementation group all were better than the control group (*P* < .05).

**Conclusion:**

Using the AIDET standard communication health education model under the King theory of goal attainment in nurse–patient communication is conducive to the rapid establishment of a harmonious and trusting nurse–patient relationship between pregnant women and nurses, helping pregnant women and nurses jointly promote the establishment and implementation of health education goals, helping to improve pregnant women’s acceptance of information related to interventional prenatal diagnosis, health education and the procedure of walking on the day of surgery. It helps enhance the effectiveness of health education and satisfaction, reducing pregnant women’s uncertainty about the disease, their unfamiliarity with the surgery environment and surgery procedure, and their preoperative anxiety.

## 1. Introduction

Birth defect refers to the abnormalities on the structural and functional metabolism of human embryos or fetuses that exist at birth, which mainly causes early miscarriage, stillbirth, perinatal death, infant death, and congenital disability, and can lead to long-term defects on children, furthermore, disability and illness seriously affect the fetuses qualities.^[1]^ According to the “China Birth Defects Prevention Report (2012)” released by the former Ministry of Health on September 2012, the current incidence of birth defects in China is about 5.6% while the number of new birth defects is about 900,000 every year.^[2]^ Amniocentesis is one of the most commonly used and safest interventional prenatal diagnosis methods on clinical practice, which plays an important role on preventing birth defects. However, due to the invasive and traumatic nature of interventional prenatal diagnosis, the lack of knowledge about interventional prenatal diagnosis and relevant health education, and the uncertainty of future unknown operations, there is uncertainty and anxiety for pregnant women before interventional prenatal diagnosis. Studies have shown that various degrees of anxiety and negative emotions, such as extreme tension and fear before surgery, can lead to endocrine and autonomic dysfunction while affecting treatment effects, postoperative recovery,^[3]^ and the increase of postoperative pain perception.^[4]^ Health education can encourage people to choose behaviors and lifestyles that are conducive to health, which is important means to promote recovery.^[5]^ Relative studies have found that patients and their families can be more confident and effectively cooperate with surgeries through fully informed effective oral or written forms about the cooperation plan including the content, the counter measures, and the information of health education, etc. It efficiently improves the surgery progress as well as decreasing the negative factors of patients such as the fear and anxiety about of operation, the surgical stress response. It has a positive impact on postoperative recovery that establishing effective communication and strengthening health education are important links.

**Figure 1.**
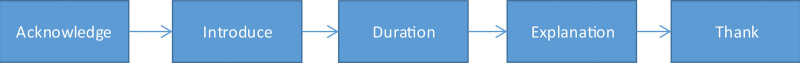
AIDET standard communication mode. It briefly explains the 5 steps of the AIDET standardized communication model.

Acknowledge (A), Introduction (I), Duration (D), Explanation (E), and Thank (T) (AIDET) communication mode, including AIDET 5 procedures, is a standardized communication mode developed by the Studer Group team. While emphasizing the importance of verbal communication and nonverbal communication, there are 5 aspects including AIDET, that promotes the procedures and standard languages of effective and in-depth communication between nurses and patient shown as Figure 1. In the actual usage of AIDET communication mode, it all achieves the better results reflected on patients satisfaction,^[6]^ the improvement of patient experience,^[7]^ the reduction of analgesic compliance,^[8]^ promoting nurse-patient relationship,^[9]^ alleviating patients anxiety,^[10]^ the harmony of nurse–patient relationship,^[11]^ increasing the quality of nursing work,^[12]^ and fully embodying the standard maintenance operation of specialist nurses and health education^[13]^, etc.

**Figure 2.**
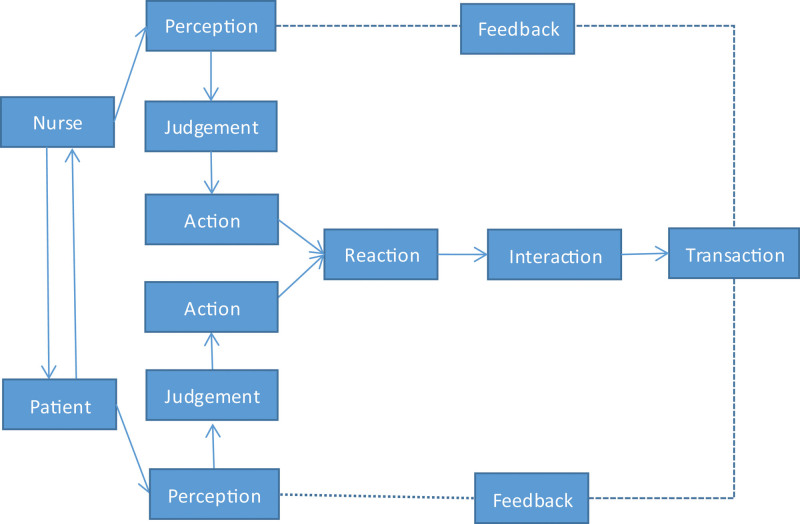
The nurse–patient interaction process of The King Theory of Goal Attainment. It briefly explains the actions, reactions and interactions between patient and nurse under The King Theory of Goal Attainment.

The King Theory of Goal Attainment shown as Figure 2, also known as the Interactive Attainment Theory, was proposed by Imo Jenny M. Kim in 1981. Its theoretical bases was Beta Langfei general system theory, which was a developed theory that conformed to the “people-oriented” medical model and put forward to a nursing theory.^[14]^ The King Theory of Goal Attainment is a systematic theory that focuses on the process of interpersonal interaction. The theory includes 4 links: interactive evaluation, the nurse–patient codetermination of achieving goals, measure implementation, and effect evaluation. It emphasizes the interaction between subject and object to conclude communication and ultimately achieve the goal, which further means nurses can preferably react on patient feelings through the perception, estimation and action of patients. The nurse–patient communication would be promoted when they have common perception and it would also have better flection back to the communicating.^[15]^ The King Theory of Goal Attainment is widely used in health education as well as achieving a relatively good effect. According to the results of existing research scholars, The King Theory of Goal Attainment is not only helping to improve patients’ awareness of disease knowledge,^[16]^ encouraging patients to actively participate in their own health maintenance,^[17]^ effective for mental health^[18]^ and the quality of life,^[19]^ etc, but also could increase the satisfaction of patients with nurses work,^[20]^ which further promote the harmony relationship between nurse and patient.^[21]^

**Figure 3.**
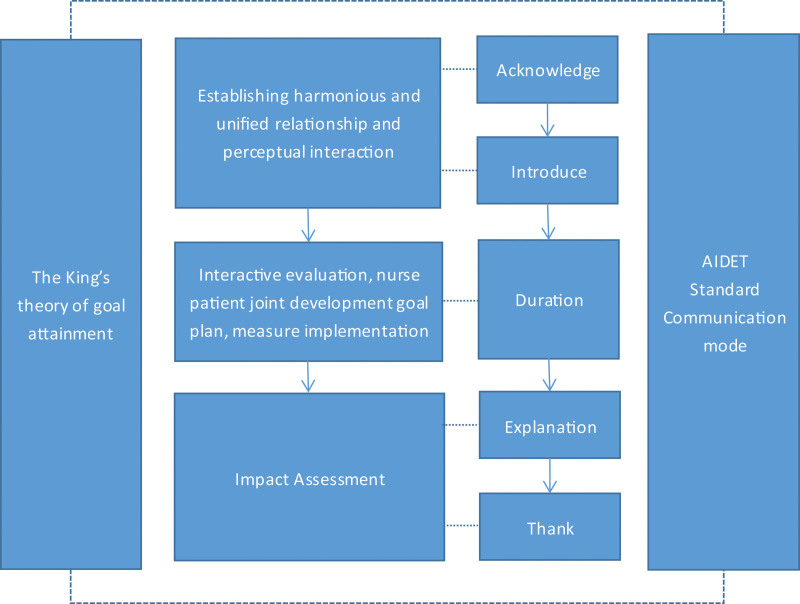
The AIDET standard communication mode under The King Theory of Goal Attainment. It briefly explains how AIDET standard communication mode and The King Theory of Goal Attainment can be interactively integrated and applied to nurse-patient communication.

After reviewing a large number of relevant literature, there is no research and report about the integration of The King Theory of Goal Attainment and AIDET communication mode, neither no implementation about outpatient communication and the outpatient health education of prenatal diagnosis care. This study aims to construct the AIDET standard communication health education mode based on The King Theory of Goal Attainment (the framework shown as Figure 3) while exploring and evaluating the actual effect of the AIDET standard communication health education mode under The King Theory of Goal Attainment in clinical application. In the actual clinical applications, the AIDET standard communication health education mode under The King Theory of Goal Attainment has achieved good results. The research results are now reported below.

## 2. Objects and Methods

### 2.1. Research object

Convenient sampling was used to select a total of 300 pregnant women who planned to undergo interventional prenatal diagnosis at the prenatal diagnosis center of a tertiary first-class hospital in Sichuan Province from the April 2020 to the January 2021, among which 150 pregnant women were randomly selected as control group who planned to undergo interventional prenatal diagnosis from the April 2020 to the August 2020 by the random number method. The rest 150 people who were about to take interventional prenatal diagnosis from the September 2020 to the January 2021 served as experimental group.

Inclusion criteria: ① Meeting the guidelines for prenatal diagnosis; ② the first time of undergoing interventional prenatal diagnosis; ③ volunteering in the study and signing the informed consent form; ④ people with self-behavior ability. Exclusion criteria: ① people with mental disorders or cognitive disorders related diseases; ② people with hearing or vision impairment; ③ unable to communicate normally. Rejection criteria: ① withdrawing from the research during the study period; ② people who have poor compliance and fail to complete the research in accordance by the intervention plan; ③ providing invalid information.

### 2.2. Research methods

#### 2.2.1. Control group.

The control group adopted the traditional communication mode: explaining the purpose and function of interventional prenatal diagnosis, indications, contraindications, limitations, risks, etc. to pregnant women as well as signing the informed consent, while making an appointment for interventional prenata and introducing the precautions before and after interventional prenatal diagnosis.

#### 2.2.2. Implementation group.

The implementation group adopted the AIDET standard communication health education mode under The King Theory of Goal Attainment on the nurse–patient communication process. The specific implementation methods are conducted below:

##### 2.2.2.1. Preparation stage.

###### 2.2.2.1.1. Establish an implementation team.

The implementation team consists of the deputy director of the nursing department, head nurses of the department, charge nurses, attending physicians, clinical nurses, health managers, and reproductive health consultants. Among them, there are 12 people including 5 senior titles, 3 intermediate titles, and 4 junior titles in total.

###### 2.2.2.1.2. Team member training.

After the team were established, the team members will be trained first. The training content includes: AIDET communication mode and The King Theory of Goal Attainment’s related concepts, frameworks and implementation examples (previous related research) and other related content. After the training, the team members will be assessed. After the team members passing the assessment, they will use the AIDET standard communication health education mode under The King Theory of Goal Attainment on the process of nurse-patient communication and implementation of health education.

##### 2.2.2.2. Implementation phase.

The AIDET communication health education mode under the King Theory of Goal Attainment are shown in Table 1.

**Table 1:** The framework of AIDET communication mode under the King Theory of Goal Attainment.

Theoretical steps	Communication procedures	Specific process	Related measures	Special attention
Establish a harmonious and unified relationship with perceive interaction	Acknowledge	Environmental preparation	Adjust the temperature, light, sound and smell of the environment	Take the initiative to greet pregnant women warmly and pay attention to keep smiling. Treat pregnant women with respect such as a sitting position, stand up to greet, slightly lower your upper body and make eye contact.
		Kind tone	Pre-inspection and triage: Please show your health code, monitor your body temperature, ask about epidemiological history, and wash your hands with anhydrous hand sanitizer. Pay attention to the tone and intonation of speaking and keep a relaxing mood	
		Take the initiative to make eye contact	Hello, this is the patient reception of the Prenatal Diagnosis Center. I am nurse XX. I am very happy to serve you and I will handle related matters for you. How can I help you?	
		Identifying	Check the diagnosis guide, payment records, name, registration number and the item barcode of the pregnant woman.	
	Introduction	Work experience	I am proficient and experienced in the work process of interventional prenatal diagnosis project appointment and health guidance.	The language of the introduction should be proficient, relaxed, and confident so that pregnant women can understand themselves through brief words, which enhances pregnant women’s trust and avoids pregnant women’s emotional tension. Meanwhile, it eliminates pregnant women’s anxiety and enhances pregnant women’s sense of dependence, security and trust.
Interactive assessment	Duration	Assess pregnant women	① Personal system: the basic information of pregnant women, understanding of interventional prenatal diagnosis, recent physical conditions (with or without abdominal pain, contractions, vaginal bleeding, etc), mental and psychological conditions (anxiety, depression, etc), and related experiments room inspection, etc. ② Interpersonal system: evaluate the interpersonal relationship and communication skills of pregnant women with family, friends, colleagues. ③ Social system: including family environment, social support situation and resources available outside the hospital, etc.	The first step of the King Theory of Goal Attainment is to conduct a comprehensive evaluation of pregnant women and guide them to tell their own problems what is needed in the process of interventional prenatal diagnosis.
Nurses and patients work together to develop a plan to achieve goals		Nurses and patients jointly set health education targets	① Interventional prenatal diagnosis (amniotic cavity/villus/umbilical blood puncture) related knowledge (including indications, contraindications, limitations, etc) ② Interventional prenatal diagnosis pregnant women surgery specifically preparations as well as pre- and postoperative precautions ③ Interventional prenatal diagnosis of pregnant women’s surgery specifically the goals to implement single use method ④ Interventional prenatal diagnosis pregnant women’s walking route and map guidance on the day of surgery ⑤ Interventional prenatal diagnosis pregnant woman walking flow chart on the day of surgery ⑥ Others (for example: the comparison of maternal blood collection, the special precautions for other test items)	According to the goals you set, what kind of methods do you want to use? Which medical staff and what content is needed to help you to understand.
		Related preparations	Informed consent form for testing items, informed consent form for amniotic cavity/umbilical blood/chorion puncture, new coronavirus epidemic notification form, ink pad, toilet paper, etc	
Measure implementation		Explain content	① Carry out targeted and individualized health education according to the health education standards established by nurses and patients: 1. Describe to pregnant women about the indications, contraindications, purpose, effects, limitations, risks, etc. of interventional prenatal diagnosis (amniotic cavity/villus/umbilical blood puncture). 2. Interventional prenatal diagnosis pregnant women surgery specifically the preparations of pre- and postoperative precautions 3. Interventional prenatal diagnosis for pregnant women with specific goals and methods in use. 4. Interventional prenatal diagnosis for pregnant women’s walking route and map guidance on the day of surgery. 5. Interventional prenatal diagnosis flow chart of walking for pregnant women on the day of surgery 6. Others (for example: the comparison of maternal blood collection, the special precautions for other test items) ② Interventional prenatal diagnosis of the common process of pregnant women on the day of surgery	After each intervention is completed, the nurse listens patiently to the pregnant woman’s thoughts: Do you think the intervention has achieved your established goals? Is there any need for you to improve? Do you have any other ideas?
Evaluation	Explanation	Emotional support and dynamic record	① Listen to pregnant women’s questions while calmly and patiently explaining them. ② According to the needs of pregnant women, provide guidance on testing and surgery related knowledge, while conducting individualized health education ③ Reevaluate the status of pregnant women and provide guidance on the main existing problems	Try to explain in a plain and easy-to-understand way when encounter professional problems and reassess the main problems of pregnant women at any time during the explanation process.
	Thank	Thanks for cooperation	① After the project is completed, the nurse actively tells the pregnant woman: Your project (such as appointment, report collection, blood collection) is completed. The nurse give the information that there is no need to save for the pregnant woman, and say: “Please keep your information”. ② Finally, express gratitude to the pregnant women, thank them for their trust, cooperation and understanding and ask them what they need to help before leaving while saying “Thank you for your cooperation.	During the entire communication process, we must pay attention to listening to the main complaints by pregnant women as well as communicating and interacting appropriately to ensure that the dialogue is relaxed, open and natural. Make pregnant women experience a warm and sincere attitude of nursing staff.

###### 2.2.2.2.1. Establish a harmonious and unified relationship and perceive interaction: Acknowledge (A), Introduction (I).

*Acknowledge*: Before communicating with pregnant women, nurse need to do the communication environment preparation well: adjusting the temperature, light, sound, smell well. When a pregnant woman comes to the patient reception of the prenatal diagnosis center, the preexamination and triage staff at the entrance greets the pregnant woman: Hello, this is the patient reception of the prenatal diagnosis center and I might measure your body temperature to know your body health status. The staff start to measure the pregnant woman’s body temperature and ask about her epidemiological history, after those steps washing hands with anhydrous hand sanitizer. When the staff communication with prenant women, need to pay attention to the tone and intonation in the process of speaking, maintaining a happy mood.

*Introduction*: When the pregnant woman entered the patient reception area, the nurse greeted warmly and introduced herself: “Hello, this is the patient reception area of the Prenatal Diagnosis Center. My name is XX as your nurse today and I am very happy to help you. I am very good at the appointment process of prenatal diagnosis and health guidance for the prenatal diagnosis. Next, I will help you make appointment for the prenatal diagnosis and give me your guidance form and payment record please, thank you.” The nurse checks the pregnant woman’s medical guide and payment records.

In the greeting and introduction link, the nurse interact with pregnant women through active and warm greetings with smiling, actively introducing themselves, creating a warm and comfortable communication atmosphere, etc so that pregnant women can reduce the impact of the unfamiliar environment around them and reduce the state of worry and anxiety of pregnant women, drawing the distance between the them and the medical staff. Therefore, it is important for pregnant woman having trust in the medical team, strengthening the pregnant woman’s sense of dependence and security, establishing a harmonious and unified doctor–patient relationship.

###### 2.2.2.2.2. Interactive assessment, nurse–patient joint development of a target plan, and implementation of measures: duration (D).

**Figure 4.**
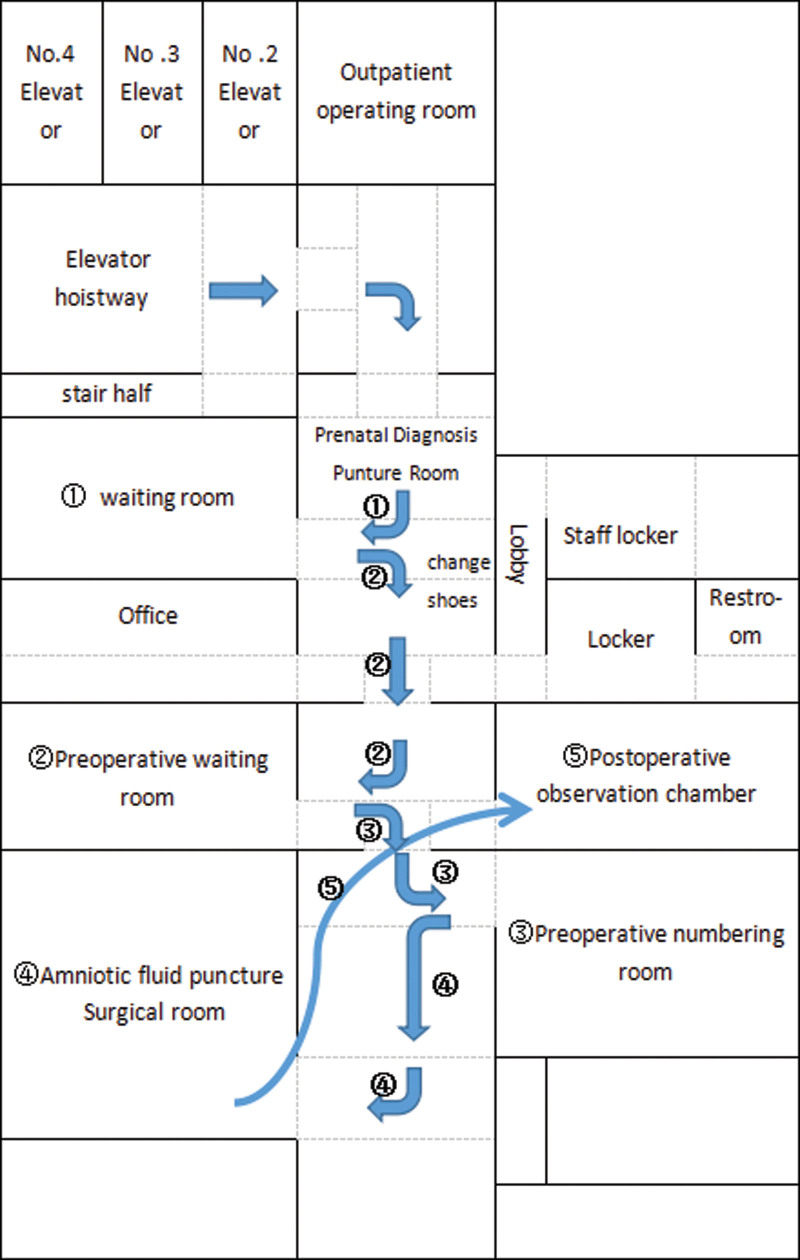
Map guidelines for interventional prenatal diagnosis of pregnant women on the day of surgery. It briefly describes how the interventional prenatal diagnosis of pregnant women will walk on the day of surgery, with a map of the walking process and destinations.

**Figure 5.**
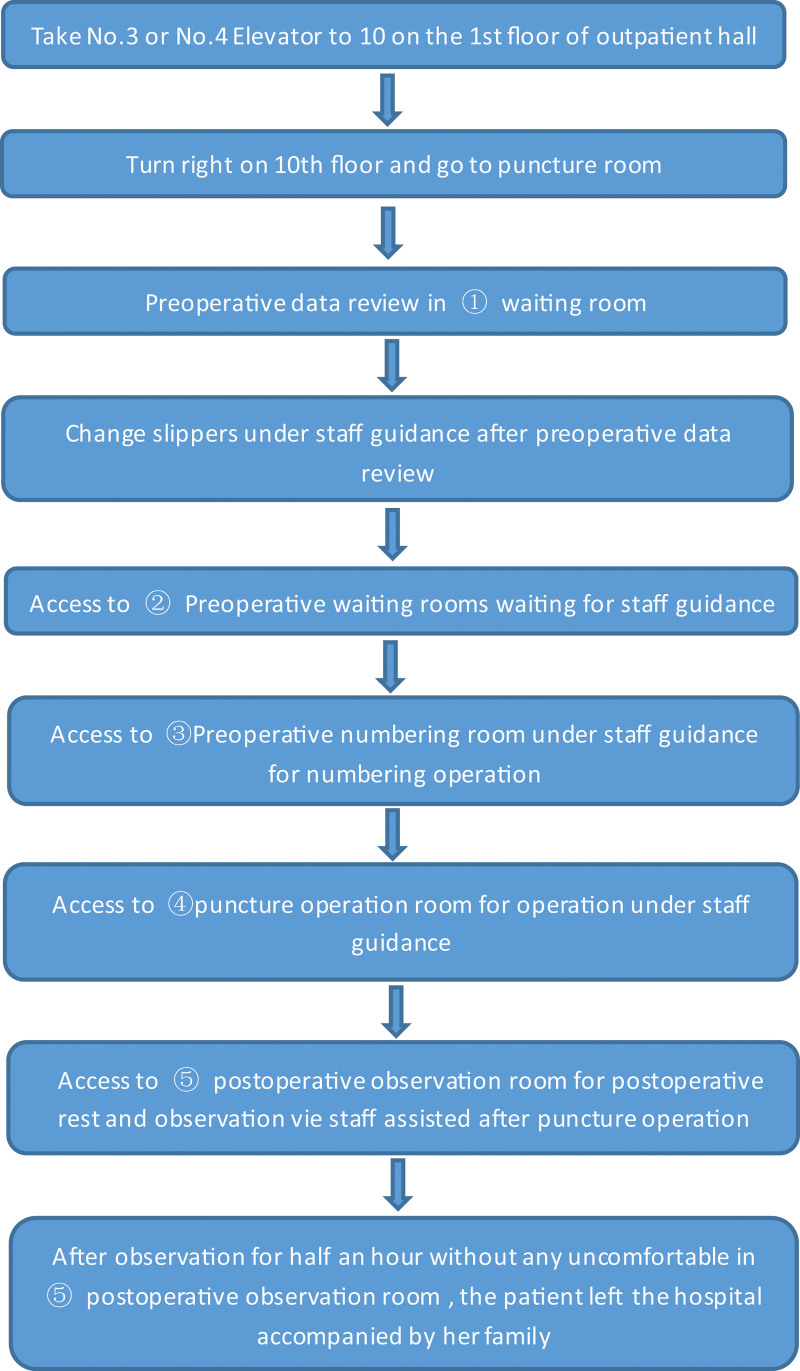
The flow chart of pregnant women walking on the day of surgery for interventional prenatal diagnosis. It briefly describes the process that the interventional prenatal diagnosis of pregnant women needs to complete on the day of surgery, with a flowchart concisely labeling the process and destination of walking.

① *Interactive evaluation—duration*: Evaluation is the first step in the process of nursing. Similarly, the King Theory of Goal Attainment emphasizes that evaluation is carried out during interaction. The content of the interactive evaluation mainly revolves around the following 3 mutually promoting systems for evaluation: (1) Personal system: the basic situation of pregnant women, which their understanding of interventional prenatal diagnosis and recent physical conditions (with or without abdominal pain, uterine contractions, Vaginal bleeding, etc) as well as mental and psychological conditions (anxiety, depression, etc) including related laboratory tests. (2) Interpersonal system: evaluating the interpersonal relationship (including family, friends, colleagues, etc) and the communication skills of pregnant women. (3) Social system: evaluating the pregnant women’s family environment, social support and the resources available outside the hospital. Nurses interact and communicate with pregnant women through the use of observation and communication skills, while assessing the current physical and psychological conditions of pregnant women. Nurses also need to understand the degree of understanding, recognition and cooperation of pregnant women with interventional prenatal diagnosis and related nursing work. Preparing for the joint development and implementation of the target plan.
② *Nursers and patients jointly develop a plan to achieve the target—duration*: Based on the information obtained from the interactive evaluation, nurses determine the current problems and influencing factors of pregnant women as well as encouraging them to take the initiative to participate. Meanwhile, nurses use the “Prenatal Diagnosis Center Health Education Target System Order” that is combined with the interventional delivery of pregnant women and the type of pre-diagnosis (amniotic cavity/umbilical cord blood/chorion puncture) to formulate the target of this health education. Both parties use their own resources to jointly seek for the best strategy to achieve the goal and finally formulate a personalized education plan. The main content of the education plan of “Prenatal Diagnosis Center Health Education Standardization Target System” includes interventional prenatal diagnosis (amniotic cavity/umbilical blood/chorion puncture) purpose, relevant indications, contraindications, limitations, risks, interventional prenatal diagnosis (amniotic cavity/umbilical cord blood/choriocentesis), pre-/postoperative precautions (diet, rest and activities, hygiene and cleanliness, etc.), interventional prenatal diagnosis, the specific goals of patients surgery methods to implement on single-use, interventional prenatal diagnosis of pregnant women’s walking route and map guidance on the day of surgery shown as Figure 4, the interventional prenatal diagnosis of pregnant women’s walking flow chart on the day of surgery shown as Figure 5, psychological adjustment and care (sense relief techniques, tension relief music), others (full exome, amniotic fluid comparison, FISH and other special attention points), etc.
③ *The measures are implemented and the interaction reaches the standard—duration*: In response to the goals jointly formulated by the “Prenatal Diagnosis Center Health Education Standardization Target System Order,” nurses provide targeted health education for pregnant women to achieve the common goals of health education implementation. According to the goal of “Prenatal Diagnosis Center Health Education Standardization Target System Order” jointly formulated with pregnant women, the following health education content is provided for pregnant women: the type of interventional prenatal diagnosis introduced for pregnant women (amniotic cavity/umbilical blood/villi puncture) to carry out the corresponding interventional prenatal diagnosis preoperative preparations and related precautions, the interventional prenatal diagnosis pregnant women surgery of the specific objectives implementation method for single use, the procedure on the day of surgery, walking route on the day of surgery, time required for surgery, corresponding precautions after interventional prenatal diagnosis, etc. For pregnant women who need psychological adjustment and care, the health manager will complete the psychological relief intervention for the them and combine the actual situation to teach them what the skills of self-regulation and tension. Also, it is suggested that the music could relieve tension and sooth emotions. It is necessary to make the environmental introduction of the amniotic fluid puncture room of the hospital and the prenatal diagnosis center. Furthermore, it is important to finish relevant laboratory tests that need to be perfected before the interventional prenatal diagnosis which is the same important as the location of the examination and the amniotic fluid puncture room of the prenatal diagnosis center, etc. The introduction of the subject development and professional level of the hospital’s prenatal diagnosis center.

###### 2.2.2.2.3. Effect evaluation: explanation (E), thank (T).

*Explanation*: All the questions and confusions raised by pregnant women need to be patiently explained. Moreover, nurses should sincerely listen to and patiently explain the questions that are queried by pregnant women and then, according to the needs of pregnant women, supply pointed testing and health education that are related to the surgery. Furthermore, the conditions of pregnant women would be reevaluate based on the existing main problems to guide.

Doctors and nurses should explain the professional queries as straightaway as possible. At the same time, the current problems should be reevaluated in the process of explanation and the trust of gravida on center staff would increase through explaining with patience to decrease gravida’s concerns and fears. Nurses should patiently listen to the patient’s thoughts: Do you think the intervention has achieved your established goals? Are there any areas for improvement? Do you have any other ideas? The nurses and patients jointly check the completion of the corresponding goals. If the goal is reached, the compliance interaction will be terminated. Otherwise, the evaluation will be conducted again. The nurses and patients will jointly analyze the reasons to adjust the plan and conduct the compliance interaction again.

*Thank*: After the project is completed, the nurse actively tells the pregnant woman: Your project is completed. Give the materials that the center don’t need to save to the pregnant woman and say: “Please keep your materials.” After the nursing service is completed, thank the pregnant women for their trust, cooperation and understanding. Meanwhile, asking them what else they need for help before leaving and say: “Thank you for your support and cooperation to our work. Can I help anything else for you?”

During the entire communication process, attention should be paid to listen for the patient’s main complaint, appropriate communication and interaction, which is necessary to ensure a relaxed, open and natural dialogue and enable pregnant women to experience the warm and sincere attitude from nursing staff.

#### 2.2.3. Research tools.

##### 2.2.3.1. General information questionnaire.

Self-made general information questionnaire that includes the pregnant woman’s name, age, gestational age, educational level, presence or absence of children, marital status, reproductive history, the history of bad pregnancy, family monthly income, residence, etc.

##### 2.2.3.2. Questionnaire on health education content of interventional prenatal diagnosis.

The self-made content questionnaire of interventional prenatal diagnosis health education is mainly based on the preoperative and postoperative precautions of interventional prenatal diagnosis as well as the procedures and walking routes on the day of interventional prenatal diagnosis. It consists of 3 levels and 12 contents: preoperative precautions for interventional prenatal diagnosis (5 items), preoperative precautions for interventional prenatal diagnosis (5 items), and procedure and walking route on the day of interventional prenatal diagnosis (2 items). In order to understand the effectiveness of health education methods and the acceptance of health education content, in this study, the reliability of the questionnaire Cronbach α coefficient is 0.905, which is indicating that the questionnaire has good reliability.

##### 2.2.3.3. Questionnaire on satisfaction of pregnant women.

Researchers have consulted a large number of literature and combined with the framework and the content of the AIDET communication health education mode under the King Theory of Goal Attainment while combining with the characteristics of the prenatal diagnosis center to summarize and design a pregnant woman satisfaction questionnaire.^[22–26]^ The satisfaction includes the following topics: “I know well about the medical staff who received me,” “I trust him/her very much,” “the medical staff who received me are very enthusiastic about helping me,” “the medical staff can fully communicate with me, during the communication, when I encounter problems,” etc. There are 10 topics in total and the questionnaire is scored by the Likert 5-level method, which is expressed according to the degree of satisfaction, that is, from “very satisfied,” “relatively satisfied,” “average,” “relatively unsatisfied,” and “very unsatisfied.” Respectively, 5-1 points are assigned and the score range is 10 to 50 points. Higher the score identifies the higher degree of satisfaction of the pregnant woman with this medical activity. In this study, the reliability of the questionnaire Cronbach α coefficient is 0.794. it is indicating that the reliability of the questionnaire is good.

##### 2.2.3.4. State Anxiety Scale (S-AI).

The State-Trait Anxiety Inventory State (S-AI)^[27]^ compiled by Spielberger and other scholars is used to measure the target’s anxiety level of pregnant women. Thereinto, the State Anxiety Inventory (S-AI) is the sub-questionnaire of STAI, which is internationally known as the gold standard for measuring anxiety levels.^[28]^ S-AI is mainly used to assess immediate or recent anxiety in a specific time state. The questionnaire consists of 20 items that half of them describe negative emotions and the rests describe positive emotions. The internal consistency reliability of the Chinese version of the scale is 0.9062 which shows good reliability and validity.^[29]^ All items in the questionnaire use Likert 4-level scoring method with the minimum score of 20 points and the maximum score of 80 points. Among them, 20 to 39 is mild anxiety, 40 to 59 means moderate anxiety, and 60 to 80 indicates severe anxiety. The S-AI score reflects the severity of the anxiety state of the research subject at the moment. The higher score shows the more severe anxiety state. The S-AI scale is widely used internationally to measure the anxiety level of patients.^[29,30]^ On this research, the Cronbach α coefficient is 0.844 and it is showing that the reliability of the questionnaire is good.

##### 2.2.3.5. Medical Uncertainty Inventory Scale (MUIS).

Using domestic scholars such as Xu Shulian^[31]^ and the 28-item translation and revision of the Medical Uncertainty Inventory Scale (MUIS) constructed and developed by the American nursing expert Mishel^[27]^ under the guidance of the uncertainty theory to form a 25-item Chinese version of the disease uncertainty scale. The disease uncertainty scale is mainly used to evaluate the 5 aspects of the patient’s symptoms, diagnosis, relationship with caregivers, treatment and prognosis,^[32]^ while including 2 dimensions of uncertainty and complexity.^[33]^ The content validity of the Chinese version of the MUIS scale is 0.920 whose the reliability coefficient is 0.865 identifies its reliability and validity are good.^[34]^ The scale uses the Likert 5-level scoring method that is from “very satisfied,” “relatively satisfied,” “average,” “relatively dissatisfied,” and “very dissatisfied,” which 5-1 points are assigned in turn with a score range of 25 to 125 points and the higher score proves the higher patient’s disease uncertainty level. Thereinto, 25 to 58.3 is divided into low level uncertainty, 58.4 to 91.7 is divided into medium level uncertainty, and 91.8 to 125 is high level uncertainty.^[35]^ The MUIS scale is widely used domestic and overseas to measure the level of uncertainty in patients’ disease.^[34,36]^ In this study, it is indicated that the reliability of the scale Cronbach α coefficient is 0.872 that the reliability of the questionnaire is good.

#### 2.2.4. Statistical methods.

SPSS25.0 software is used to establish the database for data entry and statistical analysis. The counting data is described by frequency and composition ratio and the measurement data is described by X ± S. Two-paired sample *t*-test was used for intragroup comparison before and after intervention and two-independent sample *t*-test was used for comparison between groups. X² test was used for categorical variable comparison and the test level (α) was 0.05 and the *P* value was <.05 which indicated that the difference was statistically significant.

## 3. Results

### 3.1. General information

A total number of 300 pregnant women were included in the study in which 150 were in the experimental group with an average age of 21.33 ± 3.63 years old. On the other hand, the control group concluded 150 gravidae with an average age of 21.04 ± 3.27 years old. Comparing the general data of the experimental group and the control group, the difference was not statistically significant (*P* > .05). The detailed results are shown in Table 2.

**Table 2:** Comparison of general information between the 2 groups.

Item		Implementation group	Control group	t/X^2^	*P*
Age		21.33 ± 3.63	21.04 ± 3.27	0.719*	.473
Gestational week		31.84 ± 5.49	32.46 ± 5.33	−0.992*	.322
Marital status	Unmarried	4	6	0.414†	.75
	Married	146	144		
Children	Yes	78	86	0.861†	.417
	No	72	64		
Medical insurance	Yes	123	114	1.627†	.257
	No	27	36		
Education	High school or below	56	59	0.306†	.858
	Undergraduate/college	84	83		
	Master degree or above	10	8		
Place of residence	City	91	88	0.142†	.932
	Town	34	35		
	Rural area	25	27		

* *t* value.

† X^2^ value.

### 3.2. Comparison of results of interventional prenatal diagnosis health education questionnaire

The results of the interventional prenatal diagnosis health education questionnaire in the experimental group were higher than those in the control group and the difference was statistically significant (*P* < .05). The detailed results are shown in Table 3.

**Table 3:** Comparison of the results of the interventional prenatal diagnosis health education questionnaire between the 2 groups.

Item		Implementation group	Control group	t/z	*P*
Correct number of relevant knowledge before surgery		4.88 ± 0.326	3.72 ± 0.963	13.971*	<.001
Correct rate of related knowledge before surgery		97.6 ± 6.521	74.40 ± 19.264		
Correct number of postoperative related knowledge		4.91 ± 0.292	3.74 ± 1.039	13.239*	<.001
Correct rate of postoperative knowledge		98.13 ± 5.837	74.80 ± 20.781		
Do you know the procedure on the day of surgery	Clear	143 (95.3%)	40 (26.7%)	12.095†	<.001
	Partly clear	7 (6.7%)	97 (64.7%)		
	Not clear	0 (0%)	13 (8.7%)		
Do you know the walking route on the day of surgery	Clear	147 (98%)	28 (18.7%)	13.786†	<.001
	Partly clear	3 (2%)	112 (74.7%)		
	Not clear	0 (0%)	10 (6.7%)		

* *t* value.

† *Z* value.

### 3.3. Comparison of results of pregnant women’s anxiety state and illness uncertainty

All the results of experimental group were higher than the control group and the difference was statistically significant (*P* < .05), which is shown in Table 4.

**Table 4:** Comparison of the results of the 2 groups of pregnant women’s anxiety state and illness uncertainty.

Item		Implementation group	Control group	*t*	*P*
Medical Uncertainty Inventory Scale	Total score	47.77 ± 10.451	50.99 ± 15.545	2.199	.029
	Complexity dimension	26.73 ± 6.893	28.47 ± 11.465	1.593	.112
	Uncertainty dimension	21.05 ± 5.238	22.52 ± 6.318	2.101	.036
S-AT total score		28.67 ± 6.098	35.60 ± 9.883	7.305	<.001

### 3.4. Comparison of pregnant women’s satisfaction results

All the results of experimental group were higher than the control group and the difference was statistically significant (*P* < .05), which is shown in Table 5.

**Table 5:** Comparison of the satisfaction results of the 2 groups of pregnant women.

Item	Implementation group	Control group	t	*P*
1. I knew the medical staff who received me and I trusted him/her very much	4.653 ± 0.543	4.45 ± 0.848	2.432	.016
2. The medical staff who received me were very enthusiastic about you	4.79 ± 0.442	4.43 ± 0.901	4.313	<.001
3. The medical staff who received me were very concerned about my condition and medical history as well as listening patiently	4.527 ± 0.682	4.513 ± 0.833	0.152	.88
4. The medical staff could fully communicate with me. During the communication, when I encountered a problem, I could get help from the medical staff	4.78 ± 0.447	4.49 ± 0.857	3.717	<.001
5. The medical staff who received me always gave me respect and comfort	4.8 ± 0.401	4.53 ± 0.887	3.353	<.01
6. The medical staff who received me gave me detailed information about the purpose, indications, contraindications, limitations, risks, *etc.* of the interventional prenatal diagnosis. The terms were popular and easy for me to understand.	4.77 ± 0.424	4.51 ± 0.857	3.329	<.01
7. The medical staff could tell me the information that needed to be prepared before the interventional prenatal diagnosis operation and related precautions.	4.75 ± 0.494	4.49 ± 0.88	3.073	<.01
8. The medical staff could tell me about the examinations, procedures and precautions that were needed on the day of interventional prenatal diagnosis surgery	4.77 ± 0.469	4.5 ± 0.873	3.296	<.01
9 . The medical staff could tell me the relevant precautions after the interventional prenatal diagnosis operation, the method of obtaining the report, the follow-up consultation, *etc.*	4.76 ± 0.459	4.48 ± 0.888	3.432	<.01
10. The medical staff who received me could keenly feel my needs	4.58 ± 0.678	4.46 ± 0.91	1.295	.196
Total score	47.153 ± 4.138	44.860 ± 8.255	3.042	<.01

## 4. Discussion

### 4.1. The AIDET standard communication health education mode under the King Theory of Goal Attainment improves the goal and effectiveness of health education

The results show that, from Table 3 in the 2 groups of responses to the interventional prenatal diagnosis health education questionnaire, the number of correct answers to the preoperative questions in the implementation group (4.88 ± 0.326, *P* < .001) and the correct number of post-related questions (4.91 ± 0.292, *P* < .001) were both higher than the ones of the control group (3.72 ± 0.963, *P* < .001; 3.74 ± 1.039, *P* < .001), which the difference was statistically significant. It can be considered as that the pre-/postoperative precautions Acknowledgments of pregnant women is improved through the The AIDET standard communication health education mode under the King Theory of Goal Attainment. First of all, this may be related to the positive and enthusiastic attitude of the nurse during the greetings and self-introduction, which draws the distance close between the nurse and the pregnant woman and establishes mutual trust and harmony relationship in the process of perception and interaction. The relationship lays the foundation for the follow-up interactive evaluation, nurse-patient joint development of the target plan, and the implementation of measures.

Secondly, in the process link, under the guidance of the King Theory of Goal Attainment, the process of interactive assessment, and the nurse-patient joint development of the target plan, nurses and pregnant women deepen their trust through the processes of perception, judgment, action, reaction and interaction. To the purpose of trusting each other, pregnant women can follow the nurse’s follow-up explanation and interventional prenatal diagnosis purpose, function, indications, contraindications, limitations, risks and other related information, interventional prenatal diagnosis before surgery, postoperative precautions, etc. It is accelerated that the content of health education is more valued and trusted. The nurses deepened their understanding and memory of the content through the use of common and easy-to-understand language. In addition, in the communication between pregnant women and nurses, nurses can promptly correct the problems of the pregnant women’s knowledge about interventional prenatal diagnosis, which improves the pregnant women’s awareness of interventional prenatal diagnosis. Finally, in the explanation and Acknowledgments link, the nurses evaluated the effects of the health education goals set by the pregnant women once again. If the pregnant women did not fully grasp the content of the health education goals set, the nurses would reevaluate the content that they did not master. Targeted explanations need to be multiply taught until the health education goals are fully met. Thereby, it enhanced the goals and effectiveness of health education and made health education truly change the cognition of pregnant women. It is helpful to allow pregnant women to establish a correct understanding of interventional prenatal diagnosis and to promote the purpose of pregnant women performing behaviors conducive to postoperative recovery.

Similarly, the research results once again proved that the nurses and the pregnant women have strengthened mutual trust while forming a good and harmonious nurse-patient communication relationship and cooperated with each other by implementing the AIDET standard communication health education mode under the King Theory of Goal Attainment. The improvement of concentration is promoted as well as the importance and acceptance of pregnant women for information related to interventional understanding of memory.

### 4.2 . The AIDET standard communication health education mode under the King Theory of Goal Attainment can help reduce the uncertainty about the disease and improve the preoperative anxiety of pregnant women

Pregnant women could feel intense uncertainty and anxiety about the interventional prenatal diagnosis due to that interventional prenatal diagnosis is a kind of invasive and traumatic operation, which the pregnant women receive insufficient health education and the channels to obtain relevant information are limited and incomplete. As shown in Table 4, although the scores of the implementation group and the control group for disease uncertainty and the state anxiety are in the range of the low-level of uncertainty and the mild level of anxiety. It is worthy noticed that the values of control group in these 2 categories are both critical values but this result may be more affected by the relatively lack of medical and health standards and the comprehensive quality of medical staff in other regions with relatively backward economic and health conditions.^[37]^ The implementation group’s scores of illness uncertainty (47.77 ± 10.451, *P* < .05) and state anxiety (28.67 ± 6.098, *P* < .001) are compared with the control group (50.99 ± 15.545, *P* < .05; 35.60 ± 9.883), *P* < .001) that all have different degrees of reduction, which the difference is statistically significant. Therefore, it can be considered that nurses and patients use the AIDET standard communication health education mode under the King Theory of Goal Attainment to choose the correct language to effectively communicate with pregnant women according to the standardized communication procedure. On the one hand, the standard communication mode can be used to make the nurse-pregnant woman establishing a harmonious and trusting nurse-patient relationship quickly, eliminating the sense of distance between pregnant women and nurses, and helping communicate in-depth and effective progress. On the other hand, through the standard communication mode, the nurse-patient communication can produce a homogenous effect and avoid the different communication effects caused by the different factors of the nurses themselves.

Besides, under the guidance of the King Theory of Goal Attainment, in accordance with the current individual situation of pregnant women and the individualized health education goals jointly formulated by nurses and pregnant women, pregnant women are actively invited to participate in nursing decision-making and the past passive health education methods of pregnant women are converted into active participation in the interaction with nurses through the processes of perception, judgment, action, reaction and interaction, and finally communication with each other to promote the achievement of the goal,^[38]^ which enhances the effectiveness of interaction and communication. Some scholars pointed out^[39,40]^ that the key to assessing success or failure is whether the perception of nurses and the educated object are consistent or not. The consistent perception of both nurses and patients can promote the establishment of common goals. The implementation of individualized health education enables pregnant women to fully understand their upcoming interventional prenatal diagnosis (amniotic cavity/villus/umbilical cord blood puncture) type, purpose and function, indications, contraindications, limitations, risks, and other information related to surgery, the reduction of the risk of surgery caused by information asymmetry and insufficient information, which decreases the uncertainty and anxiety. At the same time, pregnant women can fully understand that they should make correct and corresponding responses and behaviors at different times, stages, and links through obtaining targeted and individualized health education. Secondly, according to the results in Table 3, for the questions about whether acknowledging the procedure and the walking route on the day of surgery, the number of people of experimental group who were completely clear (143 people, 147 people, Z = 12.095, *P* < .001) were higher than that in the number of the control group (40 people, 28 people, Z = 13.786, *P* < .001). It can be considered that it is efficient to reduce the uncertainty, anxiety and fear influenced by the day of strange environment and the unfamiliarness of surgery content through the establishment of the operational flow chart and map guidance of interventional prenatal diagnosis, while implementing the AIDET standard communication health education mode under The King Theory of Goal Attainment. Meanwhile, it helps to reduce the uncertainty of pregnant women about the future unknown surgery process and to relieve the pregnant women’s own psychological pressure, preoperative anxiety, and their stress response. Furthermore, it also helps patients to keep a relaxed and happy state. The results are all consistent in accordance with the research of Shaha and other scholars.^[41]^

### 4.3. The AIDET standard communication health education mode under The King Theory of Goal Attainment helps to improve pregnant women’s satisfaction with medical care

As shown in Table 5, according to the satisfaction results of the 2 groups of pregnant women, it can be seen that in the experimental group “I know the medical staff who received me and I trust him/her” (4.653 ± 0.543, *P* < .05), “the medical staff who received me I am very enthusiastic about your attitude” (4.79 ± 0.442, *P* < .001), “medical staff can fully communicate with me. when I encounter problems I can get help from medical staff during the communication ” (4.78 ± 0.447, *P* < .001) “The medical staff who received me always gave me respect and comfort” (4.8 ± 0.401, *P* < .01). All 4 items were greater than the control group. This may be located in the process of implementing the AIDET standard communication health education mode under The King Theory of Goal Attainment. In the links of establishing a harmonious and unified relationship and perception interaction, the nurses take the initiative to enthusiastically welcome and accept each pregnant woman, besides the nurses take the initiative to introduce themselves to the pregnant women. The enthusiasm and active attitude from nurse also reflects respect for pregnant women. On one hand, nurses’ humanistic care for pregnant women is reflected in the entire nurse–patient communication, on the other hand, humanistic care is often reflected in a hospital’s professional and standardized health education activities. In the health education link of nurses to expectant women, at this time, nurses can often establish a good and trusting relationship with pregnant women. According to the doubts of pregnant women at this time, nurse can help and answer timely and it will help pregnant women reduce the problem of information asymmetry. Once pregnant woman have access in an unknown process or unfamiliar environment just like walked in an operation room, it will makes pregnant woman feel anxious, fear and other emotions. When nurse communicate with pregnant women by AIDET standard communication health education mode under the King theory of goal attainment, it makes pregnant woman feels that she has been valued by the nurse, at the same time, pregnant women has been respected and comforted, making the pregnant woman’s satisfaction improved.

## 5. Limitations

On the one hand, the research objects of this study are mainly selected from the prenatal diagnosis center of a tertiary hospital in Chengdu, Sichuan Province. The results could be affected by the sample size, economic development level, and comprehensive population quality. Therefore, in future research, researchers can expand the sample size and include pregnant women from the different prenatal diagnosis centers of different provinces and cities as the research objects in the study so as to avoid the impact caused by limitations in sample size, region, and population quality. In addition, there are certain obstacles in data collection, ling-term intervention and follow-up visit due to the various of samples who are mostly from multiple cities in Sichuan and a few form other provinces. Therefore, it has caused certain difficulties in carrying out long-term longitudinal research. Accordingly, in the future research, we can consider selecting local pregnant women and applying the AIDET standard communication health education mode under the King Theory of Goal Attainment, which the study process is: launching an appointment for interventional prenatal diagnosis from obstetric clinic/genetic consultation—clinic-prenatal diagnosis center-intervention—prenatal diagnosis surgery—a longitudinal study of follow-up after interventional prenatal diagnosis, in order to carry out a longer-term effect evaluation (pain perception recovery, activity recovery, complications, etc).

## Acknowledgments

The authors thank all the participants and institutions that participated in this study and gave their contribution. Thanks to Mrs. Xue Du, Mrs. Yujia Guan, Professor Wentao Peng, and Associate Professor Wanying Luo for supporting, directing, and organizing all the completed process of research.

## Author contributions

**Conceptualization:** Hao Yang, Xue Du, Yujia Guan, Wentao Peng, Wanying Luo.

**Data curation:** Wanying Luo, Yujia Guan, Wentao Peng.

**Formal analysis:** Hao Yang, Xue Du, Wanying Luo.

**Investigation:** Wanying Luo, Yujia Guan.

**Methodology:** Hao Yang, Xue Du, Yujia Guan, Wentao Peng, Wanying Luo.

**Project administration:** Hao Yang, Wentao Peng, Wanying Luo.

**Supervision:** Yujia Guan, Wentao Peng, Wanying Luo.

**Validation:** Hao Yang, Xue Du, Yujia Guan, Wentao Peng, Wanying Luo.

**Visualization:** Hao Yang, Wanying Luo, Wentao Peng.

**Writing – original draft:** Hao Yang, Wanying Luo.

**Writing – review & editing:** Hao Yang, Xue Du, Yujia Guan, Wentao Peng.
