# The implementation and effect evaluation of AIDET standard communication health education mode under the King theory of goal attainment: A randomized control study: Erratum

**DOI:** 10.1097/MD.0000000000050155

**Published:** 2026-08-14

**Authors:** Hao Yang, Wanying Luo, Xue Du, Yujia Guan, Wentao Peng

In the article “The implementation and effect evaluation of AIDET standard communication health education mode under the King theory of goal attainment: A randomized control study,”^[1]^ which appears in Volume 102, Issue 48 of *Medicine*, in Table 5, the inequality symbol in the P-value column, was incorrectly written as “>” instead of “<,” the symbol has now been corrected in the online version.
